# Genetic Diversity and Ancestral Study for Korean Native Pigs Using 60K SNP Chip

**DOI:** 10.3390/ani10050760

**Published:** 2020-04-27

**Authors:** Soo Hyun Lee, Dong Won Seo, Eun Seok Cho, Bong Hwan Choi, Yong Min Kim, Joon Ki Hong, Hyo Dong Han, Yeon Bok Jung, Dong Jun Kim, Tae Jeong Choi, Seung Hwan Lee

**Affiliations:** 1 Division of Animal and Dairy Science, Chungnam National University, Daejeon 34134, Korea; lhyungm@gmail.com (S.H.L.); seotuna@cnu.ac.kr (D.W.S.); 2 Swine Science division, National Institute of Animal Science, RDA, Seonghwan 31000, Korea; segi0486@korea.kr (E.S.C.); silveraz@korea.kr (Y.M.K.); john8604@korea.kr (J.K.H.); 3 Animal Genomics and Bioinformatics Division, National Institute of Animal Science, RDA, WanJu 55365, Korea; choibh@rda.go.kr; 4 Research and Development Division, Korea Institute for Animal Products Quality Evaluation, Areumseo-gil 21, Sejong 30100, Korea; hhdong@ekape.or.kr (H.D.H.); apgs0122@ekape.or.kr (Y.B.J.); rlaehdwns3@ekape.or.kr (D.J.K.)

**Keywords:** diversity study, ancestry, Korean native pigs, worldwide pigs, SNP, 60K Beadchip

## Abstract

**Simple Summary:**

Assessing and understanding the genetic resources of indigenous livestock populations is necessary to address issues associated with conservation and domestic supply, etc. This study examined the Korean native pig (KNP; *Sus scrofa coreanus*), which is among the native porcine breeds in South Korea, in terms of its overall genetic diversity and ancestry. According to 60K single-nucleotide polymorphism (SNP) BeadChip analyses, the KNP pig showed similarity to Western breeds than to Chinese breeds. This conclusion runs contrary to popular belief as a Chinese origin of KNP pigs, as suggested by previous historical and genetic studies. We also describe the possibility of potential biases in the analysis results.

**Abstract:**

The Korean native pig (KNP; *Sus scrofa coreanus*) is an indigenous porcine breed in South Korea considered as a valuable but dwindling genetic resource. Studies using diverse methodologies and genetic markers suggest that this population originated from the Manchu province of Northeastern China and migrated approximately 3000 years ago into the Korean peninsula. This study aimed to verify those findings by performing diversity and ancestral analyses using the 60K single-nucleotide polymorphism (SNP) BeadChip on 891 pigs of 47 breeds worldwide. We also performed principal component analysis (PCA), ancestry analyses, phylogenetic tree analysis using SNPhylo, and linkage disequilibrium analysis. Furthermore, we generated heatmap, obtained Nei’s genetic distance and *F_ST_* values, and explored the heterozygosity of commercial and native Korean pigs. The results demonstrated that KNP pigs are more closely related to European breeds than to Chinese breeds. In addition, as previous studies have suggested, our admixture analyses indicated that KNP pigs showed distinguishable genetic structure.

## 1. Introduction

Domestication of the pig took place approximately 9000 years ago [1]. Subsequently, selective breeding of pig populations has been conducted to cultivate genetic resources and to meet production requirements. Through these various challenges, efforts to discover genetic determinants of the phenotypes of superior breeds worldwide have paid off, i.e., there has been an overall increase in pork production worldwide. Genetic and genomic studies have become increasingly prominent due to advancements in breeding technology. As a priority, understanding population structure prior to the selection and mating of pigs is essential to optimize production.

The Nagoya Protocol on Access and Benefit Sharing [2] has established ethical guidelines stipulating that any financial gain resulting from the utilization of genetic resources should be shared equitably at the country level. Therefore, conservation of native domestic resources also conserves profits. Moreover, the loss of native genetic resources can be disastrous for overall genetic diversity in a given species. Any country that becomes dependent on imported seedstock due to the lack of a domestic production line may be at a financial disadvantage. Thus, studies on genetic structure are important to safeguard the genetic resources of indigenous livestock populations.

The Korean native pig (KNP; *Sus scrofa coreanus*) is, as the name suggests, one of Korea’s indigenous porcine breeds. This short-haired, black species lives in the Korean peninsula and is considered a local delicacy due to its distinctive “black coat color” pork meat. The origin of this species was first documented in the Manchu province of Northeastern China and migrated approximately 3000 years ago; it has been used for ceremonial purposes and as a source of meat throughout the Korean peninsula [3]. Recently, the meat quality of the hybrid KNP was improved by crossing with the Landrace (LR) variety [4]. Since 1960, Western breeds have been imported, marginalizing KNP in commercial meat markets due to their comparatively lower growth rates and reproductive capacity (despite their desirable sensory traits) [5,6]. In 2008, although KNP were established as purebreds and formally approved for sale at meat markets, a black-coated pig (actually a cross with Berkshire (BS) pigs) erroneously labeled as KNP was distributed, often illegally [7]. This highlighted the need to understand and evaluate the genetic structure of the KNP. For example, a study comparing the genetic structure of KNP and Western pigs used single-nucleotide polymorphism (SNP) 60K BeadChip (Illumina, San Diego, CA, USA) analyses to reveal admixture among those populations and ancestry composition as well [8]. However, SNP markers have been underutilized in assessments of the genetic diversity of KNP populations, including in comparison with other breeds worldwide because KNP is a very small indigenous population and not much used for the commercial industry. Some studies have used microsatellite markers to describe and Chinese breeds and KNP, based on which it has been suggested that the KNP population originated from Northern China [8,9,10,11]. Therefore, in order to trace the origin of KNP based on the results of previous studies, it is considered that a study is needed to trace the genetic origin by comparing the genetic components of the worldwide pig breeds.

The objective of this study is to investigate genetic migration and/or introgression among pig breeds, with a focus on KNP, using the 60K SNP BeadChip, to derive basic information on the development of pig breeds and to therefore promote the utilization and conservation of native pig breeds.

## 2. Materials and Methods 

### 2.1. Samples and Genotypes

Samples from a total of 873 pigs from 46 breeds worldwide, including KNP, were collected (Table 1). The Korean native pig samples were obtained from the National Institute of Animal Science in Korea. The international pig breed genotype datasets used in this study were provided by the Centre for Research in Agricultural Genomics (Barcelona, Spain) [12] and Jiangxi Agricultural University (Jiangxi, China) [13]. Within the datasets, the breeds were categorized by continent and region, including Africa, America (northern, southern, or central), Asia, China, and Europe. Asian regions were subsequently subdivided for the purposes of the analysis on migration and introgression events involving KNP. Genotypic data were collected using the Illumina porcine 60K SNP BeadChip. Genotype quality control was performed on the merged dataset using PLINK v1.9 software (Chang et al.; GRAIL, CA, USA) [14]. After removing 17,485 markers (call rate <0.9), the final dataset contained 34,066 SNPs. The Hardy–Weinberg equilibrium filter was not applied due to the extreme divergence seen within the dataset [15].

### 2.2. Statistical Analysis

Principal component analysis (PCA) can be performed for genetic mapping of pig breeds using eigenvectors to facilitate understanding of similarities among breeds worldwide. In this study, PCA was performed using the R package SNPRelate (Zhang et al.; University of Washington, Seattle, WA, USA) [16]. Ancestry analysis was performed using a maximum likelihood algorithm and the ADMIXTURE package [17]. Populations were characterized in terms of gene admixture based on cluster (K) analysis of ancestry after cross-validation for error estimation. Each cluster for individuals was visualized with barplot using admixed proportion. A maximum likelihood phylogenetic tree was built using the SNPhylo pipeline [18], which indicates evolutionary relationships among populations. Tree plot was illustrated using FigTree v1.4.4. The ancestry of the KNP population was analyzed at the individual chromosome level using PCAdmix (Brisbin; Cornell University, Ithaca, NY, USA) software [19]. TreeMix (Pickrell et al.; University of Chicago, Chicago, IL, USA) [20] was used to determine breeds contributing to the genetic structure of the KNP population. For linkage disequilibrium (LD) analysis, interbreeding was analyzed based on average *r^2^* values over the range 0–5000 kb. The LD results are presented by content for clarity. Based on 34,128 SNPs in KNP, the observed heterozygosity (*H_O_*) for Chinese Kele (KL), Min (MZ), and Sutai (SUT) breeds was measured and visualized at the individual chromosome level. Loss of heterozygosity over generations in each breed was measured using as the following index:

(1) Ht=H0(1−(12Ne))t

$H_{t}$, $H_{0}$, and $N_{e}$ indicate heterozygosity after t generation, heterozygosity at present generation, and effective population size calculated with LD as a *r^2^* value, respectively. Heat mapping was used to determine Nei’s genetic distances and *F_ST_* values using the R package StAMPP (Pembleton; Victoria, AU, USA) [21]. Both measurements were commonly used and differ in that Nei’s method considers mutation and genetic drift.

## 3. Results

### 3.1. Breed Diversity

**Figure 1:**
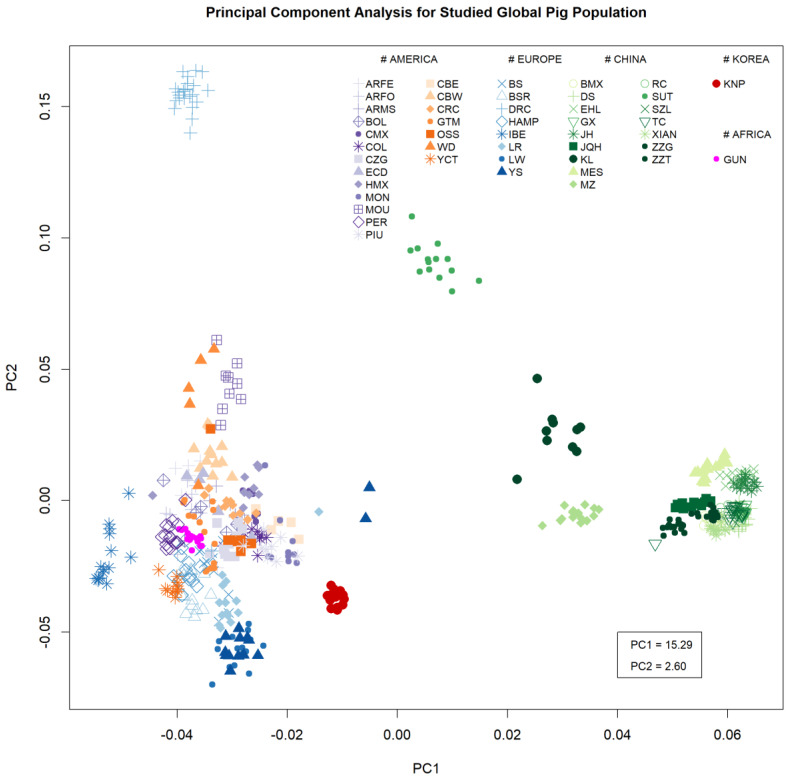
Genetic mapping based on principal component analysis: The variance explained by each component is shown in the bottom right as a percentage. (Argentina Feral (ARFE), Argentina Formosa Semi Feral (ARFO), Argentina Misiones Creole (ARMS), Bamaxiang (BMX), Bolivia Creole (BOL), Berkshire (BS), Bisaro (BSR), Cuba Eastern Creole (CBE), Cuba Western Creole (CBW), Mexico Cuino (CMX), Colombia Creole (COL), Costa Rica Creole (CRC), Colombia Zungo (CZG), Duroc (DRC), Dongshan (DS), Ecuador Creole (ECD), Erhualian (EHL), Guatemala Creole (GTM), Guinea Hog (GUN), Ganxi (GX), Hampshire (HAMP), Mexico Hairless (HMX), Iberian (IBE), Jinhua (JH), Jiangquhai (JQH), Kele (KL), Korean Native (KNP), Landrace (LR), Large White (LW), Meishan (MES), Brazil Monteiro (MON), Brazil Moura (MOU), Min (MZ), Ossabaw (OSS), Peru Creole (PER), Brazil Piau (PIU), Rongchang (RC), Sutai (SUT), Shaziling (SZL), Tongcheng (TC), White Duroc (WD), Xiang Pig (XIANG), Yucatan (YCT), Yorkshire (YS), Tibetan Gansu (ZZG), Tibetan Tibet (ZZT).)

The PCA results provided an overview of the genetic diversity of the breeds included in this study (Figure 1). Breeds were clustered at the continent or region level. In this result, the American/European cluster can be explained by first principal component, which explains 15.29% of variance, while the Asian group was clustered by second principal component, with 2.6% variance. The European population was divided by the second principal component and clustered by a few purebreds. On both edges of the second principal component, Duroc (DRC) and white coated purebreds, like LR, Large White (LW), and YS, were located. The American population comprised a large cluster of American breeds, including African guinea hog (GUN), and black-coated European breeds like BS, Hampshire (HAMP). Looking at the first principal component, the Iberian pig (IBE) ran in the opposite direction from the Chinese population and was located on the negative edge of the first component axis, close to the American population. The cluster for the KNP population (red circle) based on the first component was located close to that for the Western breeds. The KNP population was clearly isolated from other population (Figure S1).

### 3.2. Ancestry Analysis

**Figure 2:**
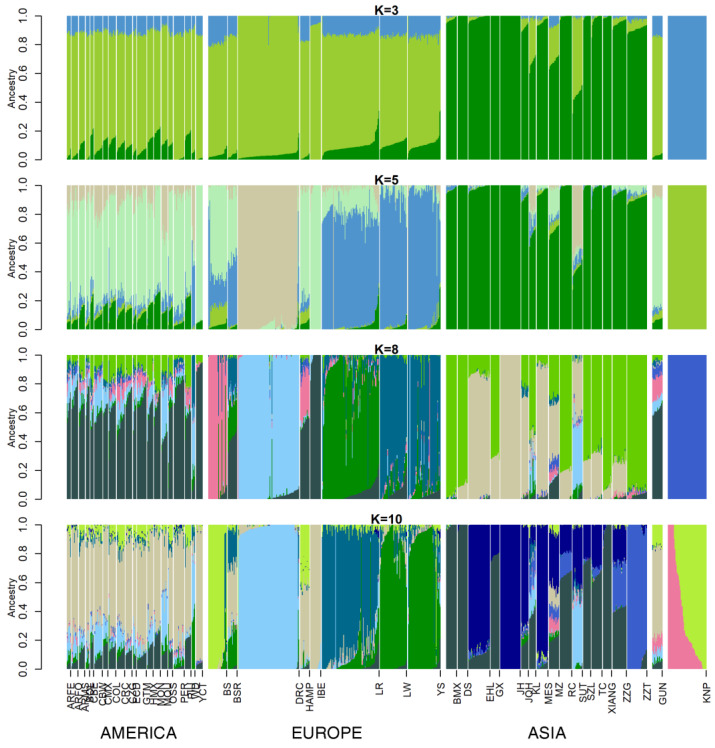
Ancestry analysis of the pig populations with admixture. (Argentina Feral (ARFE), Argentina Formosa Semi Feral (ARFO), Argentina Misiones Creole (ARMS), Bamaxiang (BMX), Bolivia Creole (BOL), Berkshire (BS), Bisaro (BSR), Cuba Eastern Creole (CBE), Cuba Western Creole (CBW), Mexico Cuino (CMX), Colombia Creole (COL), Costa Rica Creole (CRC), Colombia Zungo (CZG), Duroc (DRC), Dongshan (DS), Ecuador Creole (ECD), Erhualian (EHL), Guatemala Creole (GTM), Guinea Hog (GUN), Ganxi (GX), Hampshire (HAMP), Mexico Hairless (HMX), Iberian (IBE), Jinhua (JH), Jiangquhai (JQH), Kele (KL), Korean Native (KNP), Landrace (LR), Large White (LW), Meishan (MES), Brazil Monteiro (MON), Brazil Moura (MOU), Min (MZ), Ossabaw (OSS), Peru Creole (PER), Brazil Piau (PIU), Rongchang (RC), Sutai (SUT), Shaziling (SZL), Tongcheng (TC), White Duroc (WD), Xiang Pig (XIANG), Yucatan (YCT), Yorkshire (YS), Tibetan Gansu (ZZG), Tibetan Tibet (ZZT).)

In ancestry cluster analysis (Figure 2), 3–10 clusters were derived, where the optimal K-value was 41 (Figure S2). In detail, IBE showed a shared ancestry with all American breeds except White Duroc (WD). The degree of admixture declined with the same ancestry in IBE, with increasing ancestry number. DRC showed a partially shared ancestry with the American breeds and with SUT. Chinese breeds shared a similar ancestry, except for KL, MZ, and SUT. KL and MZ showed a partially shared ancestry with American and European breeds when 8–10-cluster solutions were used. SUT had a shared ancestry with DRC at all K-values. For KNP, no admixture was seen at K-values of levels of 3–8. KNP shared very few genetic components with others at 5–10 clusters. At a K-value of 10, KNP shared very few genetic components with HAMP, MZ, or most of the American breeds and shared a lot with BS. When the K-value was 41, the optimal number of ancestries, KNP consisted mainly of three ancestry mixtures, with averages of 0.377, 0.308, and 0.314. These three-ancestry proportions scored higher than 0.1 in the KNP population, whereas it was hardly detected in others except Cuba Western Creole (CBW) in one ancestry (0.11). Results with only Asian population depicted in Figure S3. 

**Figure 3:**
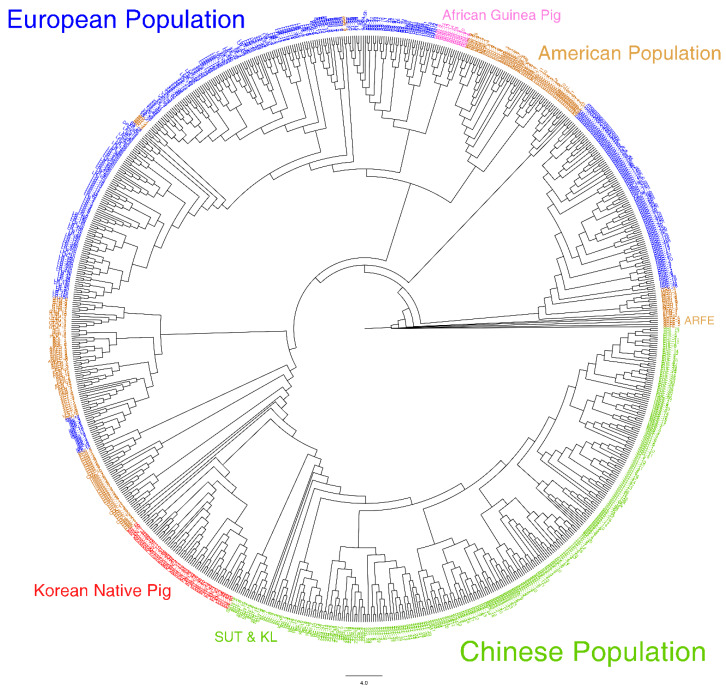
Maximum likelihood phylogenetic tree (created using SNPhlyo) of the pig populations in this study. (Argentina Feral (ARFE), Argentina Formosa Semi Feral (ARFO), Argentina Misiones Creole (ARMS), Bamaxiang (BMX), Bolivia Creole (BOL), Berkshire (BS), Bisaro (BSR), Cuba Eastern Creole (CBE), Cuba Western Creole (CBW), Mexico Cuino (CMX), Colombia Creole (COL), Costa Rica Creole (CRC), Colombia Zungo (CZG), Duroc (DRC), Dongshan (DS), Ecuador Creole (ECD), Erhualian (EHL), Guatemala Creole (GTM), Guinea Hog (GUN), Ganxi (GX), Hampshire (HAMP), Mexico Hairless (HMX), Iberian (IBE), Jinhua (JH), Jiangquhai (JQH), Kele (KL), Korean Native (KNP), Landrace (LR), Large White (LW), Meishan (MES), Brazil Monteiro (MON), Brazil Moura (MOU), Min (MZ), Ossabaw (OSS), Peru Creole (PER), Brazil Piau (PIU), Rongchang (RC), Sutai (SUT), Shaziling (SZL), Tongcheng (TC), White Duroc (WD), Xiang Pig (XIANG), Yucatan (YCT), Yorkshire (YS), Tibetan Gansu (ZZG), Tibetan Tibet (ZZT).)

Phylogenetic tree using maximum likelihood started with Argentina Feral (ARFE) (Figure 3). African Guinea Pigs (GUN) were surrounded in the western population branch. KNP was located in the end branch of the western population and start point of the Eastern population. SUT led the top branch in the Chinese population and followed by KL. Asian population tree depicted in Figure S4.

**Figure 4:**
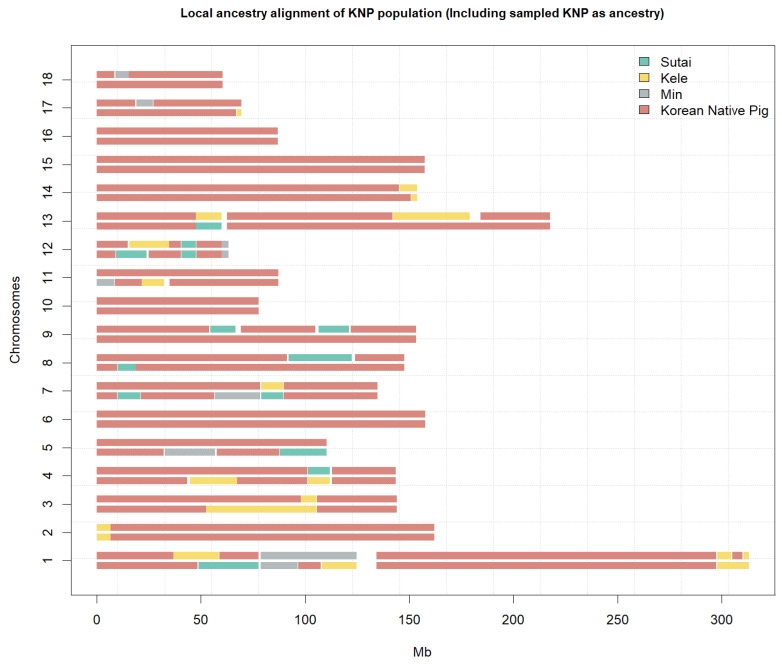
The local ancestry of the KNP, analyzed using the PCAdmix program: Several KNP populations were included in the ancestry analysis to verify the lineage of the KNP population. Sutai (SUT), Kele (KL), and MZ were included by TreeMix as branches of the phylogenetic tree of KNP (Figure S6).

KL, MZ, and SUT were included by TreeMix as branches of the phylogenetic tree of KNP. In Figure 4, KNPs were included as local ancestry to verify indigenous patterns among the ancestries selected with TreeMix. As a result, those ancestral windows with KL, MZ, and SUT were observed very little. Additionally, in Figure S5, where only Chinese ancestry was considered, KL was the most dispersed ancestry in entire windows. This local ancestry occupied all of chromosome 3 and 9. Most of the windows showed diploidically similar length and ancestry.

### 3.3. Genetic Diversity

The *H_O_*, expected heterozygosity (*H_S_*), and inbreeding coefficient (*F_IS_*) values for each breed are shown in Table 1. The *F_IS_* value, which ranges from −1 to 1, illustrates the degree to which a target population is inbred, where positive value indicates a high degree of inbreeding. The Asian population ranged from 0.153 to 0.363 for *H_O_* and 0.122 to 0.283 for *H_S_*; *F_IS_* ranged from −0.243 to −0.148. The mean *F_IS_* values were −0.192, −0.201, and −0.194 in the American, Asian, and European populations, respectively. For the KNP population, *F_IS_*, *H_O_*, and *H_S_* had values of −0.233, 0.214, and 0.167, respectively.

**Figure 5:**
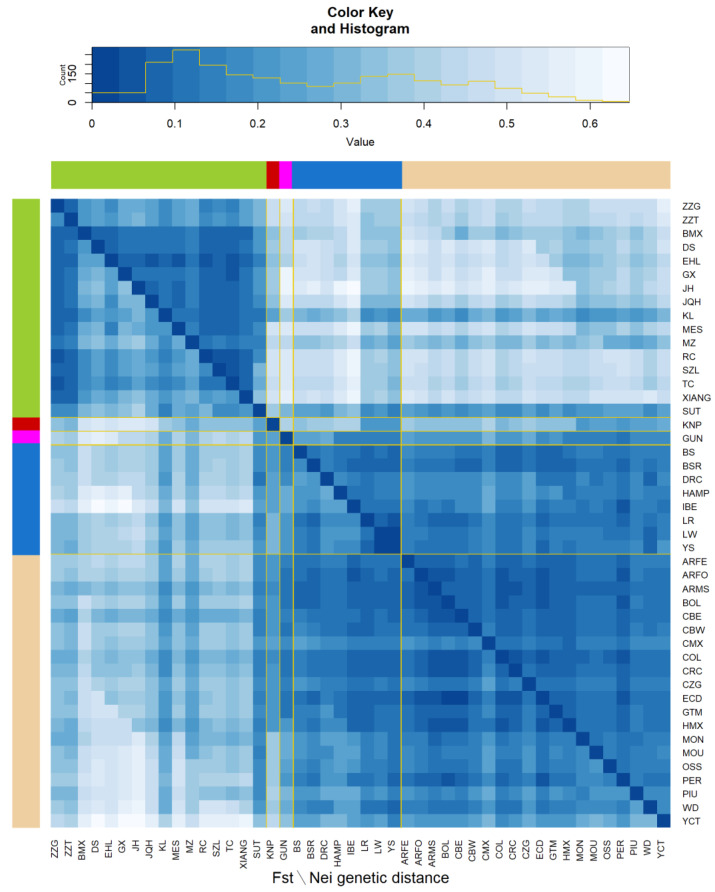
Heat map used to derive F_ST_ and Nei’s genetic distance values for the Chinese populations (olive green), KNP (red), American guinea hog (magenta), European populations (blue), and American populations (off-white). (Argentina Feral (ARFE), Argentina Formosa Semi Feral (ARFO), Argentina Misiones Creole (ARMS), Bamaxiang (BMX), Bolivia Creole (BOL), Berkshire (BS), Bisaro (BSR), Cuba Eastern Creole (CBE), Cuba Western Creole (CBW), Mexico Cuino (CMX), Colombia Creole (COL), Costa Rica Creole (CRC), Colombia Zungo (CZG), Duroc (DRC), Dongshan (DS), Ecuador Creole (ECD), Erhualian (EHL), Guatemala Creole (GTM), Guinea Hog (GUN), Ganxi (GX), Hampshire (HAMP), Mexico Hairless (HMX), Iberian (IBE), Jinhua (JH), Jiangquhai (JQH), Kele (KL), Korean Native (KNP), Landrace (LR), Large White (LW), Meishan (MES), Brazil Monteiro (MON), Brazil Moura (MOU), Min (MZ), Ossabaw (OSS), Peru Creole (PER), Brazil Piau (PIU), Rongchang (RC), Sutai (SUT), Shaziling (SZL), Tongcheng (TC), White Duroc (WD), Xiang Pig (XIANG), Yucatan (YCT), Yorkshire (YS), Tibetan Gansu (ZZG), Tibetan Tibet (ZZT).)

In the heat map (Figure 5), lower and upper diagonal parts indicate the Nei’s genetic distance and *F_ST_* values for each population. Regions are distinguished by color. Most of the breeds were clustered into Eastern and Western clusters. The Asian breeds KNP, SUT, KL, and MZ showed similarity to the Western breeds and had average *F_ST_* and Nei’s genetic distance values of 0.302 and 0.218, respectively. The *F_ST_* and Nei’s distance values for KNP with respect to the American/European population were 0.323 and 0.253, respectively, while those with respect to the Chinese population were 0.378 and 0.409, respectively. The overall *F_ST_* and Nei’s genetic distance values for KNP were 0.345 and 0.317, respectively.

### 3.4. Linkage Disequilibrium and Observed Heterozygosity

**Figure 6:**
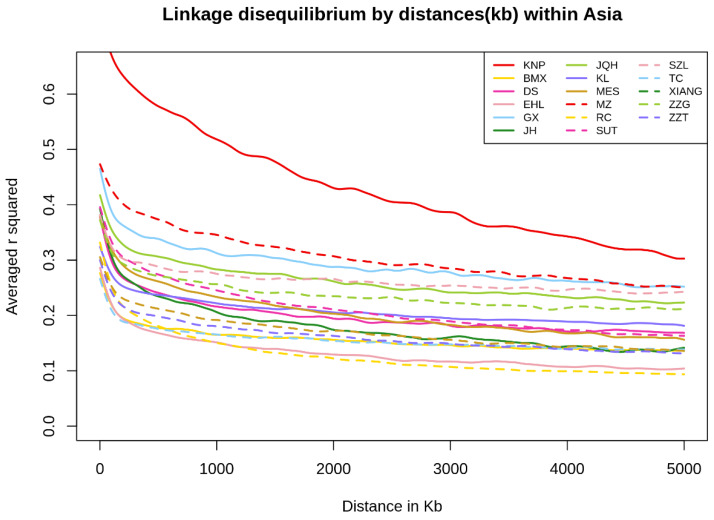
Average *r^2^* values for the Asian breeds: The *Y*-axis range was limited to 0–0.65 for easy comparison of the linkage disequilibrium (LD) values among all breeds. (Bamaxiang (BMX), Dongshan (DS), Erhualian (EHL), Ganxi (GX), Jinhua (JH), Jiangquhai (JQH), Kele (KL), Korean Native (KNP), Meishan (MES), Min (MZ), Rongchang (RC), Sutai (SUT), Shaziling (SZL), Tongcheng (TC), Xiang Pig (XIANG), Tibetan Gansu (ZZG) and Tibetan Tibet (ZZT).)

LD analyzed based on average *r^2^* values for each region and breed (Figure 6, Figures S7 and S8). The *Y*-axis range was limited to 0–0.65 for easy comparison of the values among regions. KNP, MZ, and Ganxi (GX) showed high LD values (average *r^2^* 5000 Kb = 0.33, 0.27, and 0.25, respectively). Regarding the European and African populations (Figures S7 and S8), GUN had the largest *r^2^* value, followed by HAMP and Bisaro (BSR) (average *r^2^* 5000 Kb = 0.29, 0.14, and 0.18, respectively). Finally, Mexican Cuino (CMX) and WD had *r^2^* values of 0.37 and 0.33, respectively.

**Figure 7:**
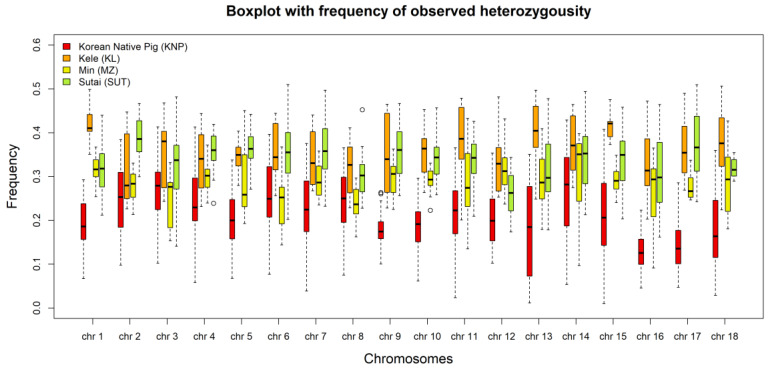
Box plot of *H_O_* values for the KNP, KL, MZ, and SUT breeds by chromosome.

Based on TreeMix, the *H_O_* was depicted with boxplot for KNP, KL, MZ, and SUT. Figure 7 shows that the average *H_O_* was less than 0.26 for KNP while, for KL, it exceeded 0.32 for all chromosomes. KL was the most heterogeneous breed with respect to chromosome 1, with a value of 0.42. The KNP population showed the lowest average *H_O_* value for chromosome 18 of 0.18. 

**Figure 8:**
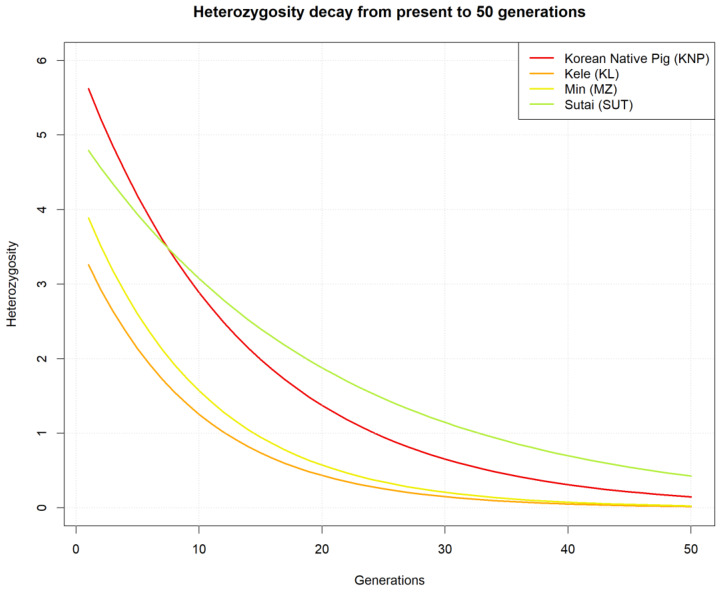
Changes in heterozygosity within 50 generations for the KNP, KL, MZ, and SUT breeds.

Figure 8 shows the change in degree of heterozygosity by generation for each population, based on the present effective population size and heterozygote frequency. For the KNP, the value decreased from almost 5.4 over 50 generations, corresponding to a non-heterozygous population; this negative inclination was the largest among all of the breeds studied herein.

## 4. Discussion

Historical records show that KNP migrated from Manchu province in China to Korea [3]. This record understood the origin of the KNP to be that Chinese wild boars migrated to Korea and were domesticated into KNP in Jeju island. However, as a result of analyzing the mitochondrial D-loop sequence, it was confirmed that Chinese wild boars were transmitted to Korea after they were domesticated contrary to what was previously known [22]. In addition, previous studies that compared KNP with Western and Chinese pig breeds based on microsatellite markers suggested that KNP originated from the Chinese black-coated pig and subsequently interbred with Western breeds [11]. Furthermore, those studies noted low genetic distance of KNP, which clustered with MZ pigs according to PCA and phylogenetic tree analyses. In consideration of those results, this study illuminated some genetic characteristic with an ancestry (admixture) analysis, for which the K-value was 8; KNP showed a slight similarity to HAMP, BS, MZ, and several American pig breeds (see Figure 2; the ancestry of KNP is indicated in blue). According to our SNPhylo results, the KNP population shares branches with black-coated pigs and Chinese pig populations. In addition, the heat map of genetic distance indicated that the KNP was farther away from the Chinese population compared to the Western population. The *F_ST_* and Nei’s genetic distance values were 0.496 and 0.306, respectively, for KNP with respect to the Chinese populations; the values with respect to the Western populations were 0.359 and 0.219, respectively.

The differences in results between this study and those of previous studies of KNP may be explained as follows. First, as also previously mentioned, northern Chinese breeds migrated to the Korea peninsula in the distant past, whereas European genes were introduced to the Korean pig populations in the 20th century. Second, BeadChip was designed for application to commercial pig breeds from Europe and America [23,24,25], such that results related to Asian pigs may be biased given the low number of Asian pig-related polymorphism. Thus, we assumed that the analyses of Asian pig breeds performed in this study may have been subject to ascertainment bias [24,26]. The large gap between those two events presents unique challenges for genetic analyses; mating between KNP and European breeds occurred much later than that between KNP and Chinese breeds. The KNP population was more likely to experience dramatic allele replacement following the introduction of Western breeds. 

Whole-genome sequencing (WGS) of KNP and commercial breeds have also been studied to characterize genetic variation therein; numerous novel SNPs were found in each population, which further underscores the need for up-to-date swine SNP arrays [9]. Although this previous research was mainly concerned with genome partitioning, the results revealed that at least some KNP had a partially shared ancestry with commercial breeds. Meanwhile, the SNP analyses in this study showed that the KNP had an ancestry largely free from admixture. The discrepancy in the results implies that the information used to develop the commercial 60K SNP arrays can have different coverage from WGS, which could lead to bias depending on the method used by a given study.

Inbreeding among KNP populations was suggested by the LD results; the LD was high compared to that of other breeds based on the average *r^2^* value (see Figure 6 and Figure 7 and Table 1). The number of LD combinations for KNP (1,406,530) was not larger than that for SUT (4,163,179), while 100% (*r^2^* = 1) LD combined markers were 189,751 (KNP), and 64,065 (SUT). Due to the ratio of complete LD markers to overall LD markers, some breeds, such as WB and KNP, had high levels of LD in entire distances, as compared to others, in addition to high LD levels in short distances, implying that KNP maintained its genetic integrity for many generations, similar to many aboriginal species. Though high-LD block pruning was applied, homologous diploid patterns, in many windows, through LD filtering, reflects that the KNP may occasionally be homozygotic (see Figure S5). In other words, paternal and maternal windows with the same ancestry and same window size may depict homologous genetic states. The *H_O_* and the changes in heterozygosity over generations (see Figure 7 and Figure 8, respectively) show the gradual shift in KNP toward homogeneity. Based on the TreeMix results, it appears that, after being separated from KL, MZ, and SUT by humans, KNP mated only with other KNP individuals from within for many generations. 

The dendrogram generated using TreeMix shows a slightly different ancestry compared to the SNPhylo results. In the TreeMix dendrogram, the root node corresponds to Western populations and branches out to Chinese populations (SUT, MZ, and KL). KNP is in the middle of the dendrogram. This pattern was also observed when KNP was added as ancestry (see Figure 4), implying that the segregation of KNP populations from Chinese populations occurred a long time ago. In addition, compared with previous studies, this result may indicate that the origin of the KNP is related to these three populations [11,27]. The KNP has confirmed the high relationship with domesticated Chinese wild boars [22], and Korean wild boar was highly related to the Chinese pig breed of XIANG [11]. Moreover, the genetic distances of MZ pigs and KLs were confirmed close and XIANG (each 0.85 and 0.613) pig had relatively far distances with those two populations [27]. Although these results are difficult to compare directly, we can estimate the relationship between these populations. However, these estimates need to be verified with a comparative study of many samples and accurate genotypes.

## 5. Conclusions

In this study, the KNP population showed an authentic genetic profile among all of the porcine populations studied, especially in the admixed pattern. This population had an intermediate position in the PCA plot, more kin to Western breeds, and was located among Western and Asian breeds in the phylogenetic tree analysis. In addition, according to the phylogenetic tree and a genetic distance heat map, KNP was closer to the Western breeds than the Chinese breeds. This is estimated to be the result of the crossing of European pig breed before the 1930s. Since the KNP has maintained a small group with small breeding since it was introduced to Korea, the average heterozygosity value (*H_O_* and *H_S_*) and *F_IS_* value are estimated to be lower than most of the breeds. These results are believed to have long maintained indigenous pig breeds of KNP since they entered Korea, but we can assume crossbreeding can also be seen. Therefore, it is necessary that more research to accurately identify the genetic structure will be required for the genetic improvement of KNP.

## Figures and Tables

**Table 1:** Characteristics of the pigs used in this study, stratified by continent.

Population	Abbr.	Number of Samples	Continent (Country)	*F_IS_*	*H_O_*	*H_S_*
Guinea Hog	GUN	15	Africa	−0.21561	0.24466	0.19339
Bamaxiang	BMX	16	China	−0.18743	0.21812	0.17437
Dongshan	DS	15	China	−0.22474	0.19643	0.15194
Erhualian	EHL	32	China	−0.15704	0.19786	0.16120
Ganxi	GX	13	China	−0.24343	0.17913	0.13651
Jinhua	JH	30	China	−0.19250	0.15328	0.12247
Jiangquhai	JQH	11	China	−0.20828	0.22652	0.17738
Kele	KL	10	China	−0.22069	0.36251	0.28308
Meishan	MES	17	China	−0.14848	0.17325	0.14384
Min	MZ	15	China	−0.24263	0.28690	0.21950
Rongchang	RC	18	China	−0.17866	0.20472	0.16476
Sutai	SUT	15	China	−0.21308	0.33809	0.26723
Shaziling	SZL	11	China	−0.23253	0.21988	0.16979
Tongcheng	TC	16	China	−0.16408	0.22455	0.18138
Xiang Pig	XIANG	13	China	−0.19884	0.19039	0.14982
Tibetan Gansu	ZZG	21	China	−0.18170	0.22380	0.17871
Tibetan Tibet	ZZT	29	China	−0.19512	0.24058	0.19119
Korean Native Pig	KNP	57	Korea	−0.23283	0.21350	0.16689
Berkshire	BS	28	Europe	−0.19111	0.30093	0.24058
Bisaro	BSR	14	Europe	−0.20780	0.32414	0.25618
Duroc	DRC	91	Europe	−0.17946	0.28352	0.22941
Hampshire	HAMP	14	Europe	−0.17519	0.24523	0.19857
Iberian	IBE	16	Europe	−0.14276	0.19132	0.15988
Landrace	LR	85	Europe	−0.20920	0.34154	0.27423
Large White	LW	40	Europe	−0.19803	0.32323	0.26175
Yorkshire	YS	49	Europe	−0.22586	0.36596	0.28915
Cuba Eastern Creole	CBE	5	Central America	−0.19443	0.36501	0.29197
Cuba Western Creole	CBW	12	Central America	−0.20699	0.33331	0.26401
Costa Rica Creole	CRC	12	Central America	−0.15263	0.27830	0.23168
Guatemala Creole	GTM	14	Central America	−0.18924	0.29959	0.24041
Ossabaw	OSS	7	North America	−0.18876	0.29628	0.23614
White Duroc	WD	5	North America	−0.23209	0.35621	0.27499
Yucatan	YCT	10	North America	−0.19945	0.21675	0.17234
Argentina Feral	ARFE	6	South America	−0.16744	0.28001	0.22843
Argentina Formosa Semi Feral	ARFO	10	South America	−0.16979	0.29542	0.24152
Argentina Misiones Creole	ARMS	9	South America	−0.19796	0.34920	0.27903
Bolivia Creole	BOL	6	South America	−0.14305	0.26133	0.21780
Colombia Creole	COL	11	South America	−0.18893	0.33441	0.26967
Colombia Zungo	CZG	10	South America	−0.22178	0.31298	0.24301
Ecuador Creole	ECD	5	South America	−0.17857	0.32963	0.26671
Mexico Cuino	CMX	7	South America	−0.26775	0.32025	0.24199
Mexico Hairless	HMX	9	South America	−0.17319	0.30347	0.24695
Peru Creole	PER	16	South America	−0.18019	0.28900	0.23336
Brazil Monteiro	MON	10	South America	−0.14992	0.27048	0.22507
Brazil Moura	MOU	9	South America	−0.21217	0.32382	0.25486
Brazil Piau	PIU	9	South America	−0.21873	0.28493	0.22212

*F_IS_* (Inbreeding Coefficient), *H_O_* (frequency of Observed heterozygosity), and *H_S_* (frequency of Expected heterozygosity).

## Supplementary Materials

The following are available online at https://www.mdpi.com/2076-2615/10/5/760/s1, Figure S1: Genetic mapping based on principal component analysis with 3 dimensional, Figure S2: Cross-validation for error estimation, performed before the ancestry (K) cluster analysis, Figure S3: Ancestry analysis conducted on the Asian pig populations using the ADMIXTURE package, Figure S4: Maximum likelihood phylogenetic tree (created using SNPhlyo) of the Asian pig populations, Figure S5: Ancestry analysis of the KNP population performed using the PCAdmix program, Figure S6: TreeMix analysis to select breeds for inclusion in the KNP ancestry analysis, Figure S7: Average *r^2^* values for the American pig breeds. The *Y*-axis range was limited to 0–0.65, for easy comparison of the LD values of all breeds, Figure S8: Average *r^2^* values for the European and African pig breeds. The *Y*-axis values were limited to 0–0.65, for easy comparison of the LD values of all breeds.
