# Supplementary material for: Genetic Diversity and Ancestral Study for Korean Native Pigs Using 60K SNP Chip
Source: Animals (Basel). 2020 Apr 27;10(5):760. doi: 10.3390/ani10050760 (PMC7277343; doi:10.3390/ani10050760)
Supplement: Supplementary file 1 [file animals-10-00760-s001.zip › animals-734912-Supplementary Materials.docx]

**Supplementary Materials:**


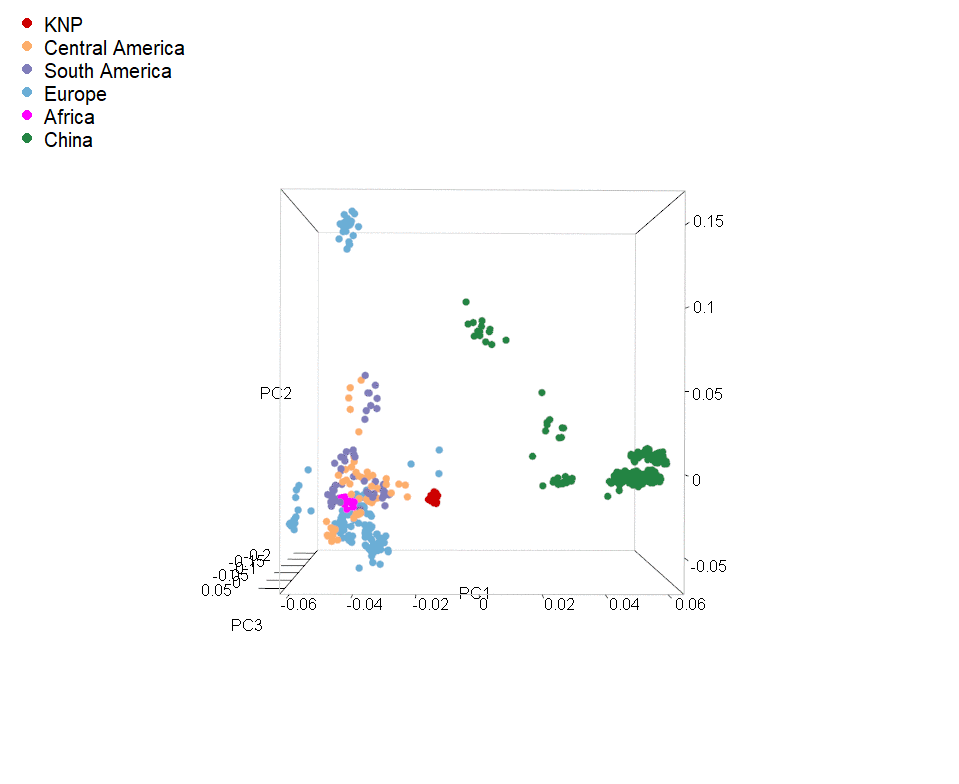


**Figure S1.** Genetic mapping based on principal component analysis with 3 dimensions.


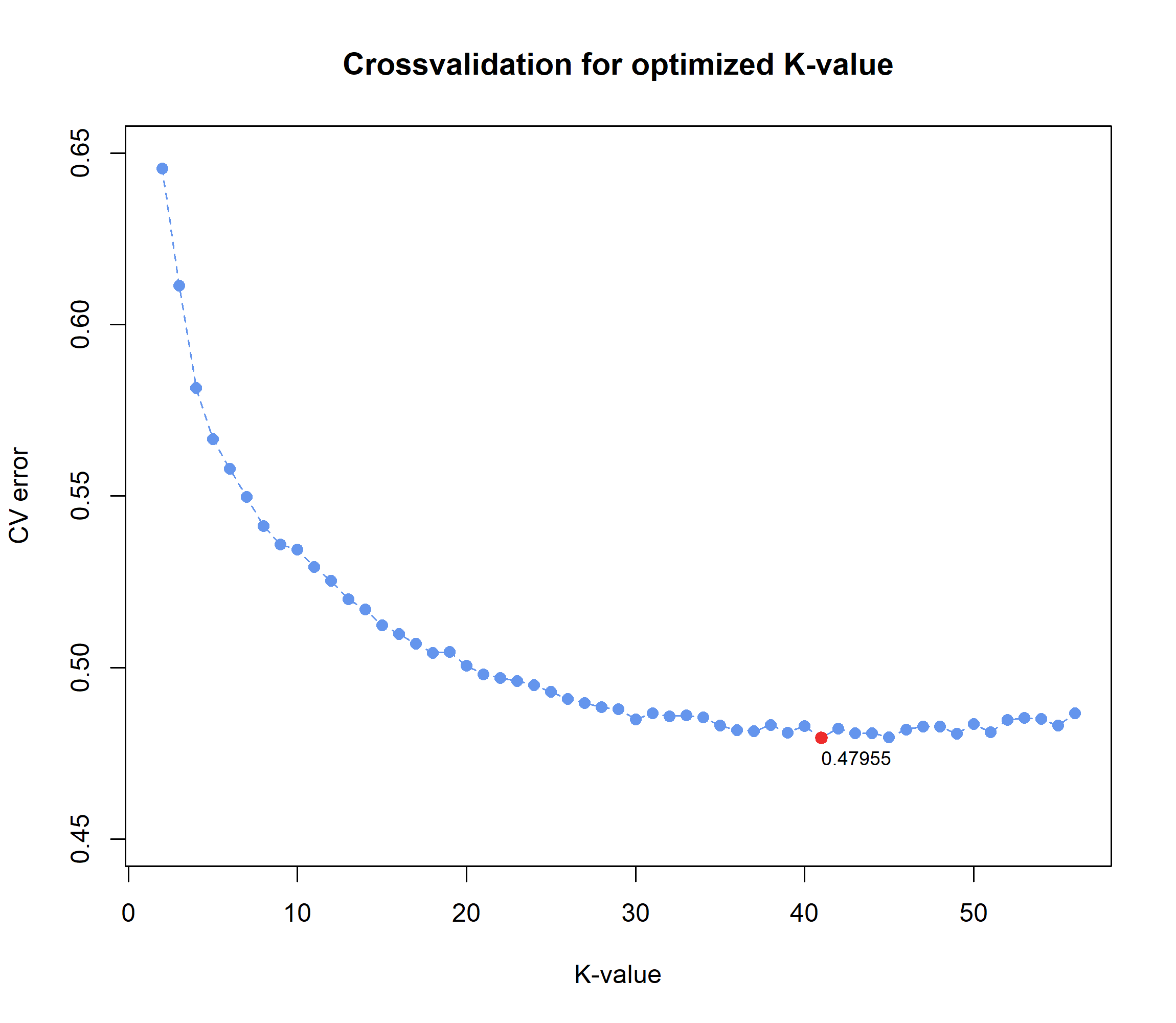


**Figure *2.*** Cross-validation for error estimation, performed before the ancestry (K) cluster analysis. The optimum K value was 0.47955 (K = 41) for 873 pigs (46 breeds).


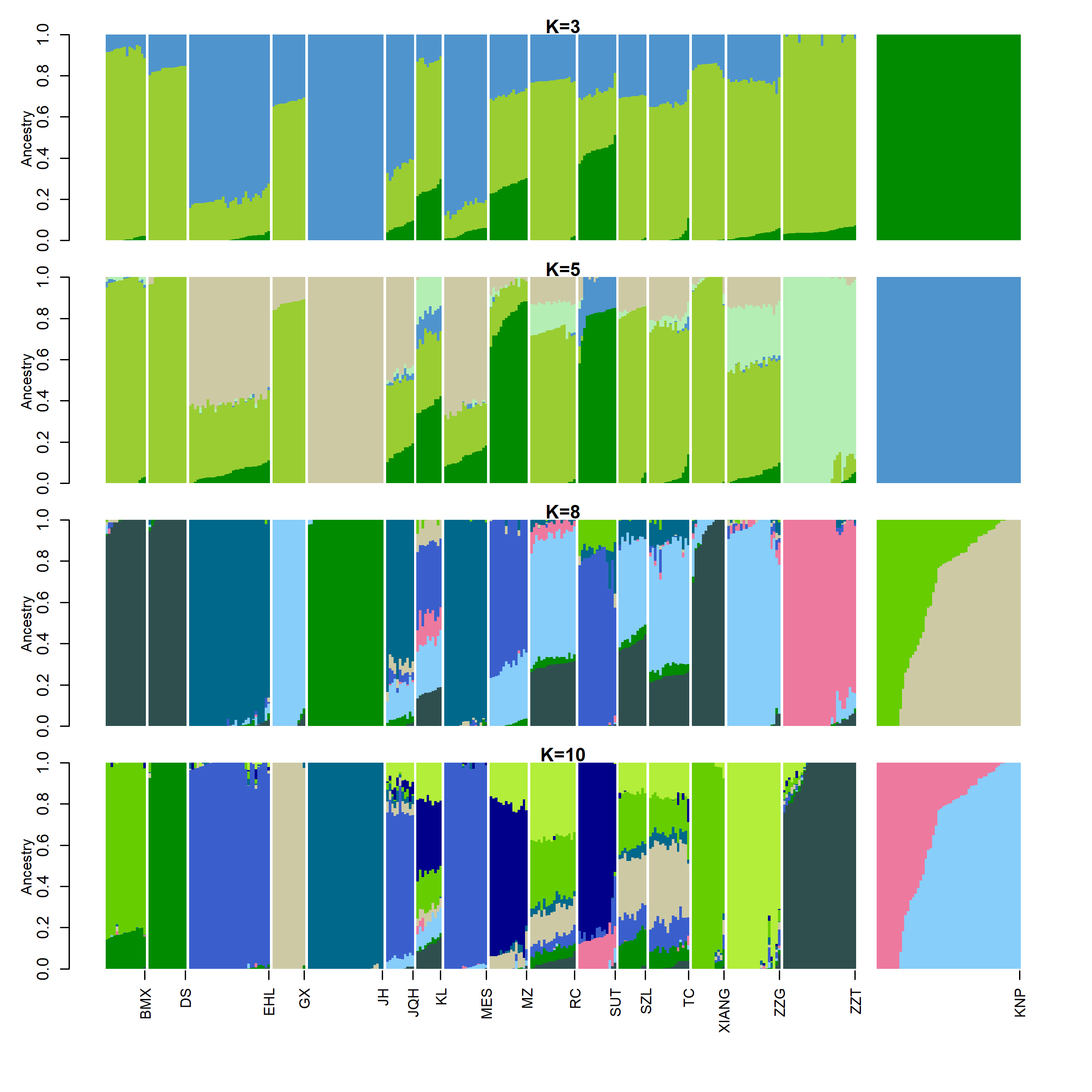


**Figure S3.** Ancestry analysis conducted on the Asian pig populations using the ADMIXTURE package. Bamaxiang (BMX), Dongshan (DS), Erhualian (EHL), Ganxi (GX), Jinhua (JH), Jiangquhai (JQH), Kele (KL), Korean Native (KNP), Min (MZ), Rongchang (RC), Sutai (SUT), Shaziling (SZL), Tongcheng (TC), Chinese Wild Boar (WB), Xiang Pig (XIANG), Tibetan Gansu (ZZG), Tibetan Tibet (ZZT).


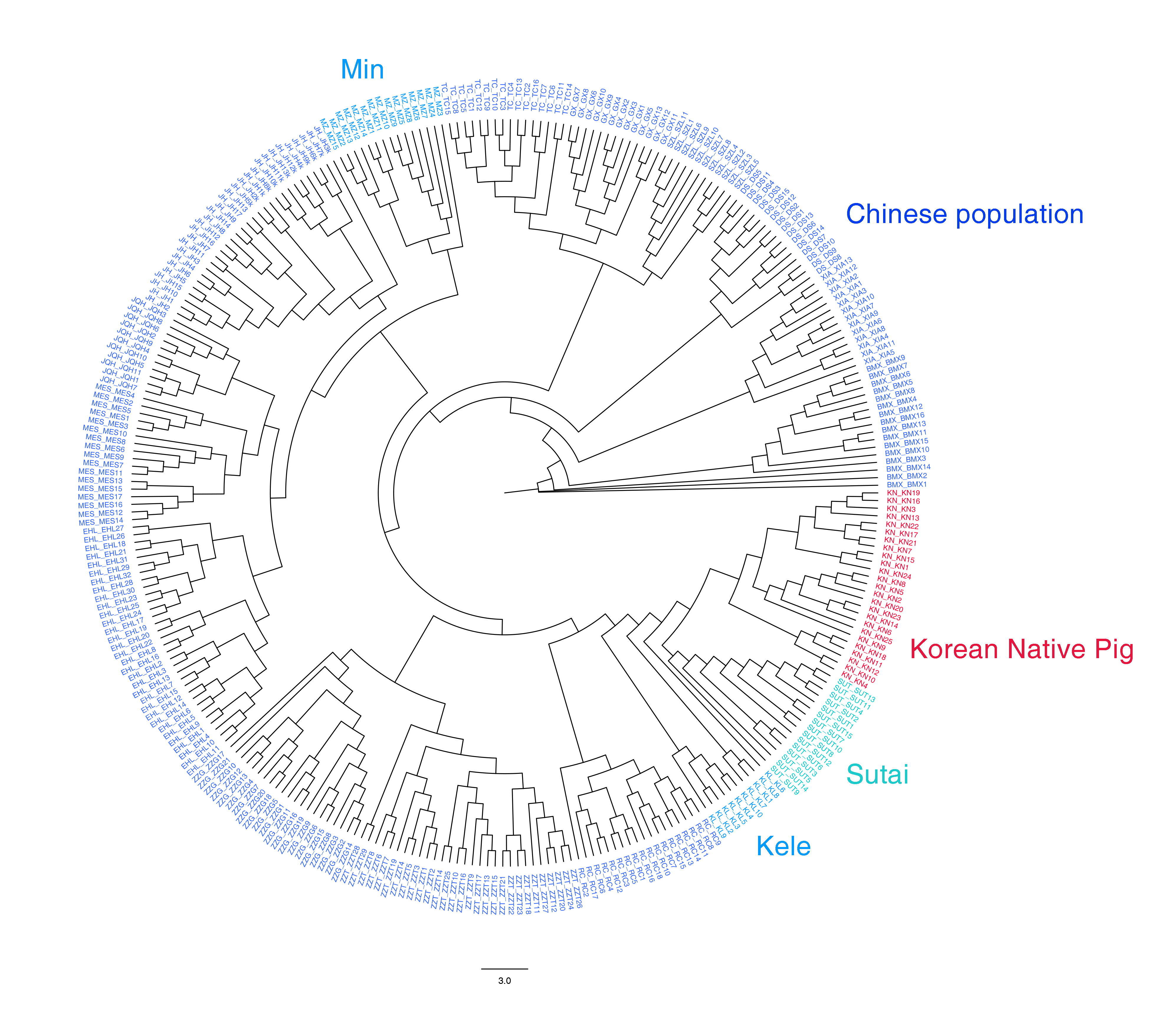


**Figure S4.** Maximum likelihood phylogenetic tree (created using SNPhlyo) of the Asian pig populations. Bamaxiang (BMX), Dongshan (DS), Erhualian (EHL), Ganxi (GX), Jinhua (JH), Jiangquhai (JQH), Kele (KL), Korean Native (KNP), Min (MZ), Rongchang (RC), Sutai (SUT), Shaziling (SZL), Tongcheng (TC), Xiang Pig (XIANG), Tibetan Gansu (ZZG), Tibetan Tibet (ZZT).


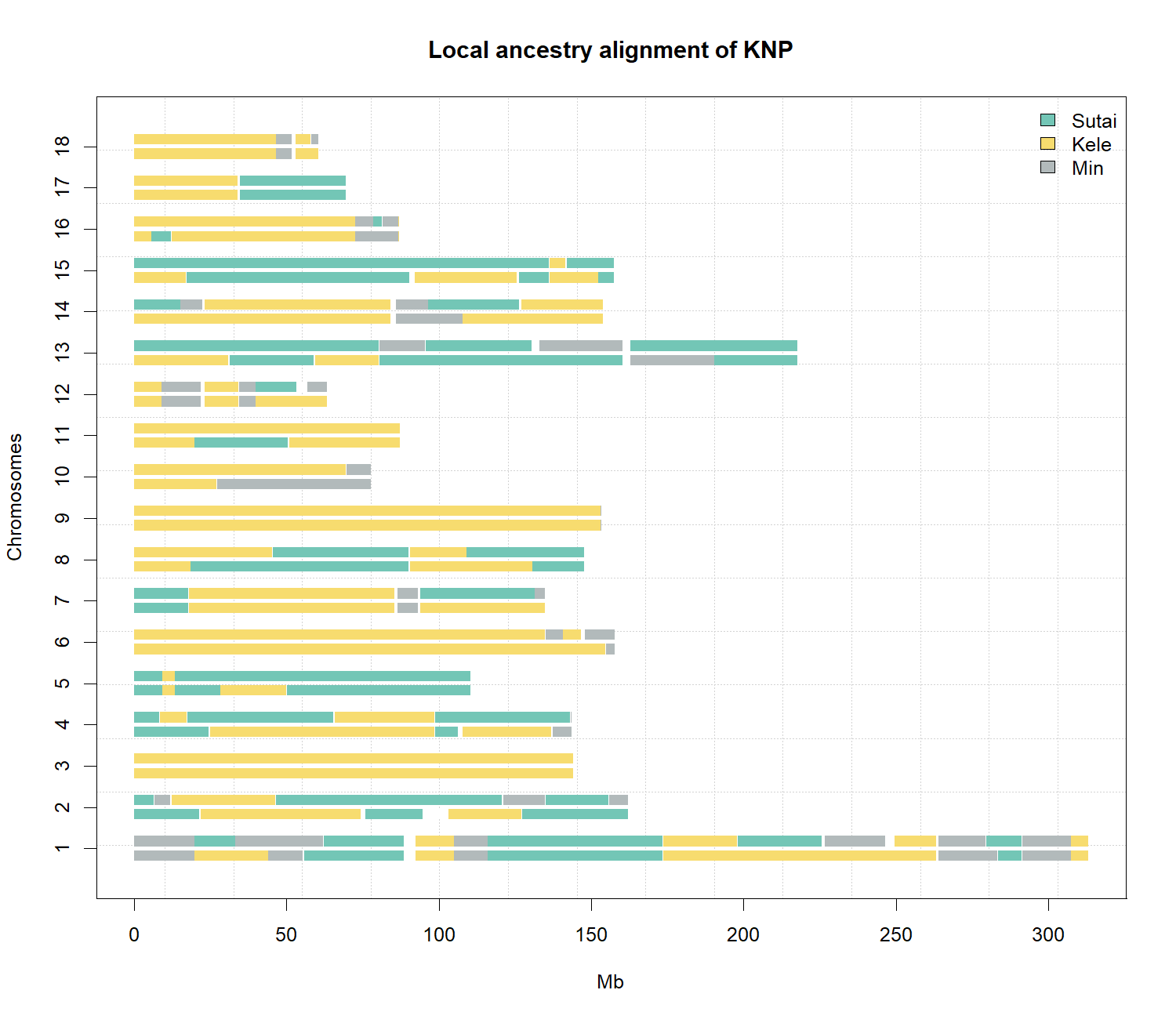


**Figure S5.** Ancestry analysis of the KNP population performed using the PCAdmix program. The Chinese breeds SUT, KL, and MZ were selected based on the TreeMix analysis described in Figure S6.


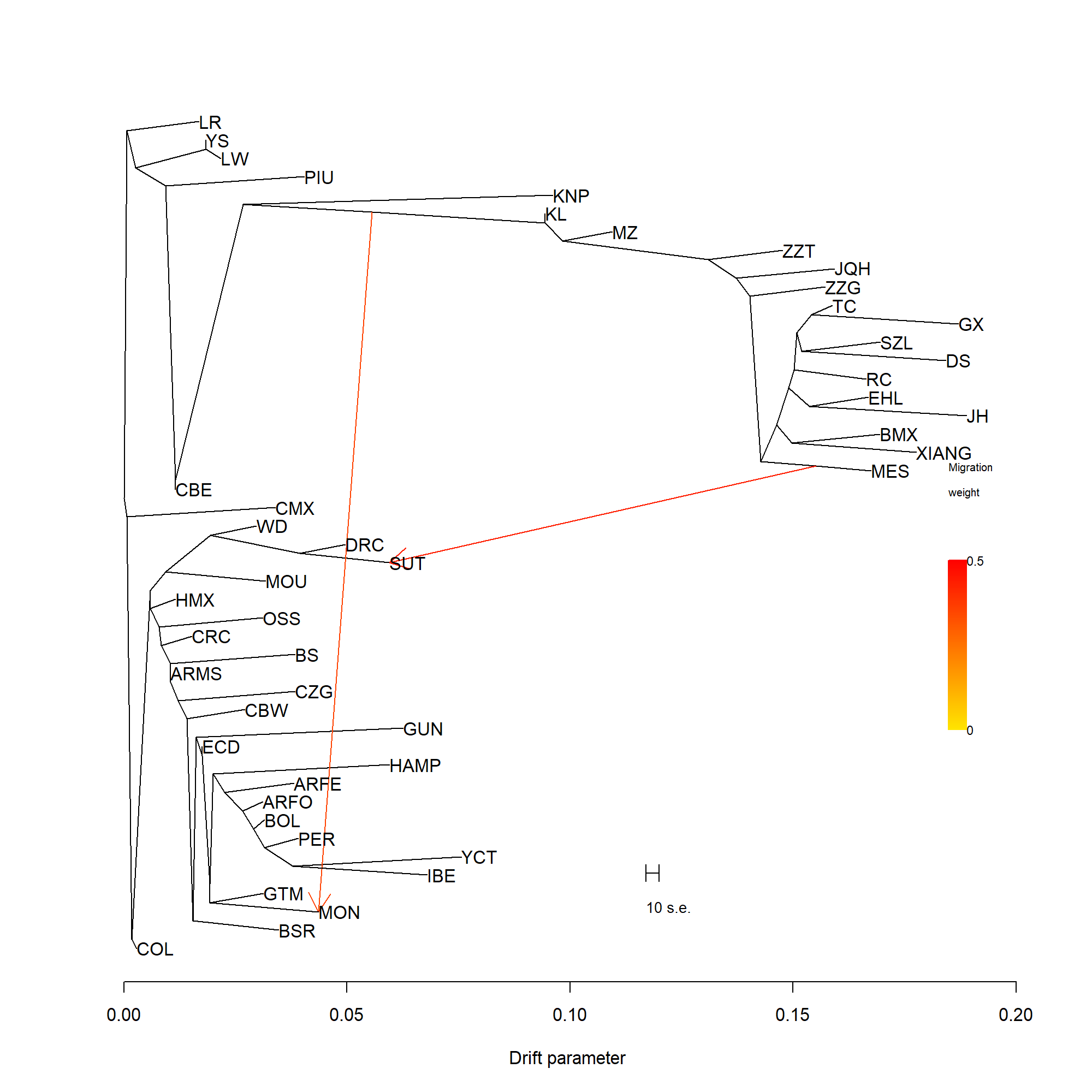


**Figure S6.** TreeMix analysis to select breeds for inclusion in the KNP ancestry analysis. Argentina Feral (ARFE), Argentina Formosa Semi Feral (ARFO), Argentina Misiones Creole (ARMS), Bamaxiang (BMX), Bolivia Creole (BOL), Berkshire (BS), Bisaro (BSR), Cuba Eastern Creole (CBE), Cuba Western Creole (CBW), Mexico Cuino (CMX), Colombia Creole (COL), Costa Rica Creole (CRC), Colombia Zungo (CZG), Duroc (DRC), Dongshan (DS), Ecuador Creole (ECD), Erhualian (EHL), Guatemala Creole (GTM), Guinea Hog (GUN), Ganxi (GX), Hampshire (HAMP), Mexico Hairless (HMX), Iberian (IBE), Jinhua (JH), Jiangquhai (JQH), Kele (KL), Korean Native (KNP), Landrace (LR), Large White (LW), Meishan (MES), Brazil Monteiro (MON), Brazil Moura (MOU), Min (MZ), Ossabaw (OSS), Peru Creole (PER), Brazil Piau (PIU), Rongchang (RC), Sutai (SUT), Shaziling (SZL), Tongcheng (TC), White Duroc (WD), Xiang Pig (XIANG), Yucatan (YCT), Yorkshire (YS), Tibetan Gansu (ZZG), Tibetan Tibet (ZZT).


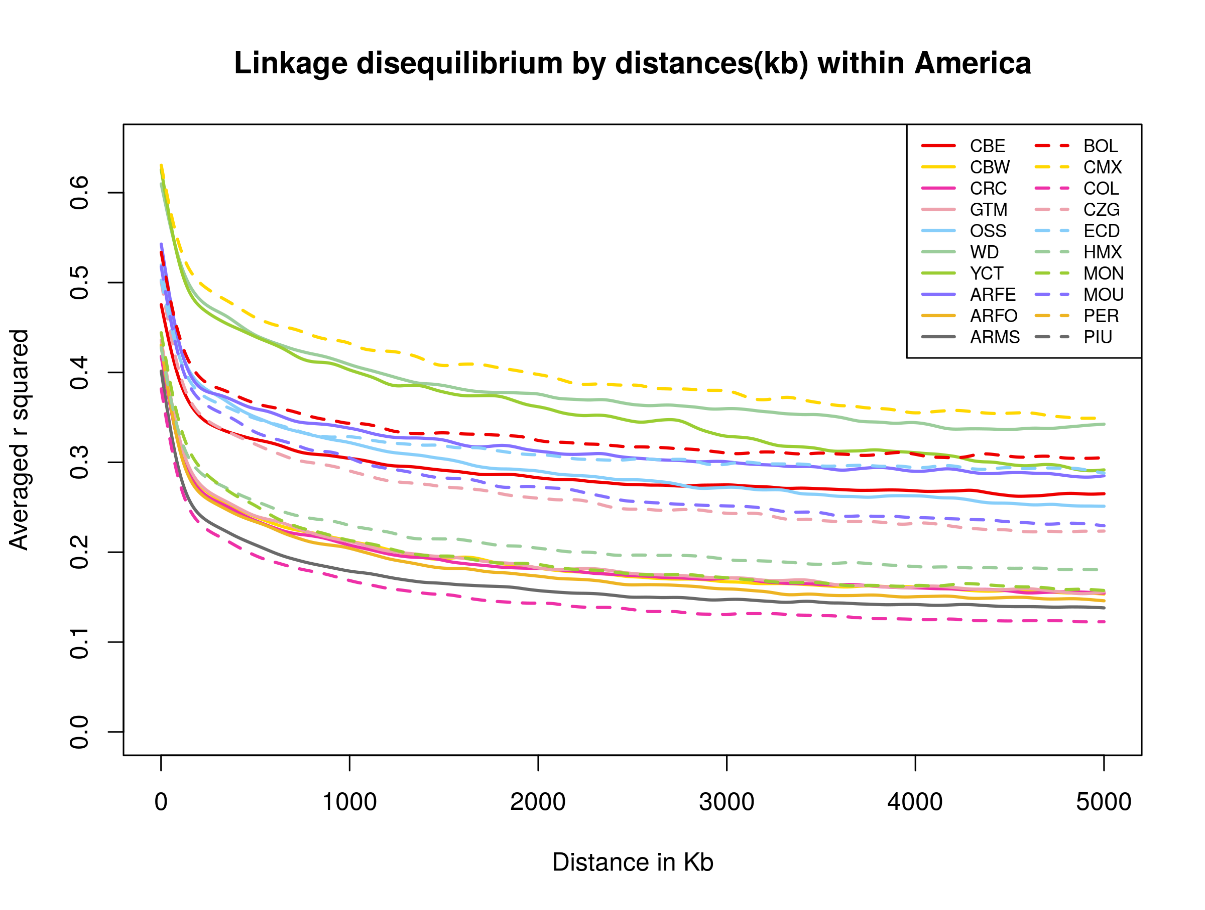


**Figure S7.** Average *r^2^* values for the American pig breeds. The Y-axis range was limited to 0–0.65, for easy comparison of the LD values of all breeds. Argentina Feral (ARFE), Argentina Formosa Semi Feral (ARFO), Argentina Misiones Creole (ARMS), Bolivia Creole (BOL), Cuba Eastern Creole (CBE), Cuba Western Creole (CBW), Mexico Cuino (CMX), Colombia Creole (COL), Costa Rica Creole (CRC), Colombia Zungo (CZG), Ecuador Creole (ECD), Guatemala Creole (GTM), Mexico Hairless (HMX), Brazil Monteiro (MON), Brazil Moura (MOU), Ossabaw (OSS), Peru Creole (PER), Brazil Piau (PIU), White Duroc (WD) and Yucatan (YCT).


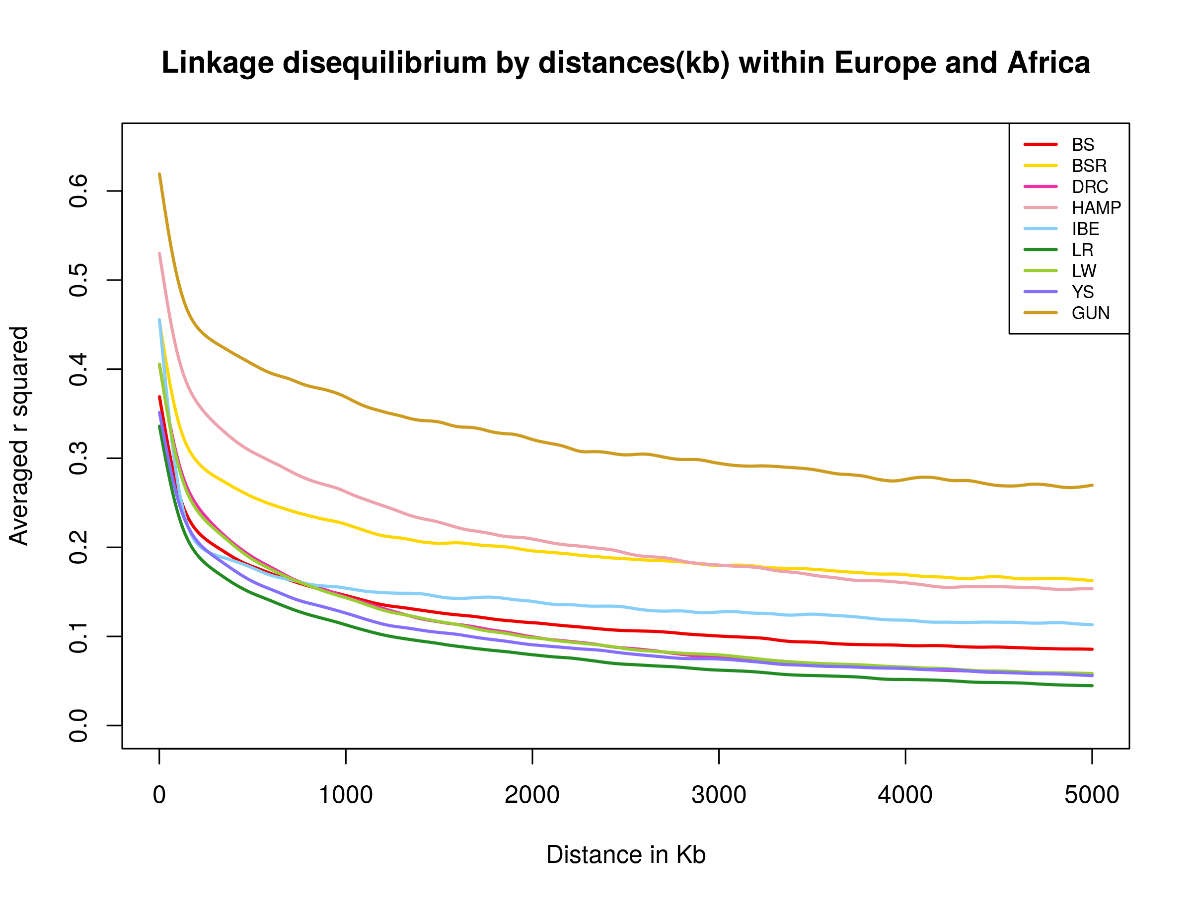


**Figure S8.** Average r^2^ values for the European and African pig breeds. The Y-axis values were limited to 0–0.65, for easy comparison of the LD values of all breeds. Berkshire (BS), Bisaro (BSR), Duroc (DRC), Guinea Hog (GUN), Hampshire (HAMP), Iberian (IBE), Landrace (LR), Large White (LW) and Yorkshire (YS)*.*
