# Supplementary figures and images for: Genetic Diversity and Ancestral Study for Korean Native Pigs Using 60K SNP Chip
Source: Animals (Basel). 2020 Apr 27;10(5):760. doi: 10.3390/ani10050760 (PMC7277343; doi:10.3390/ani10050760)

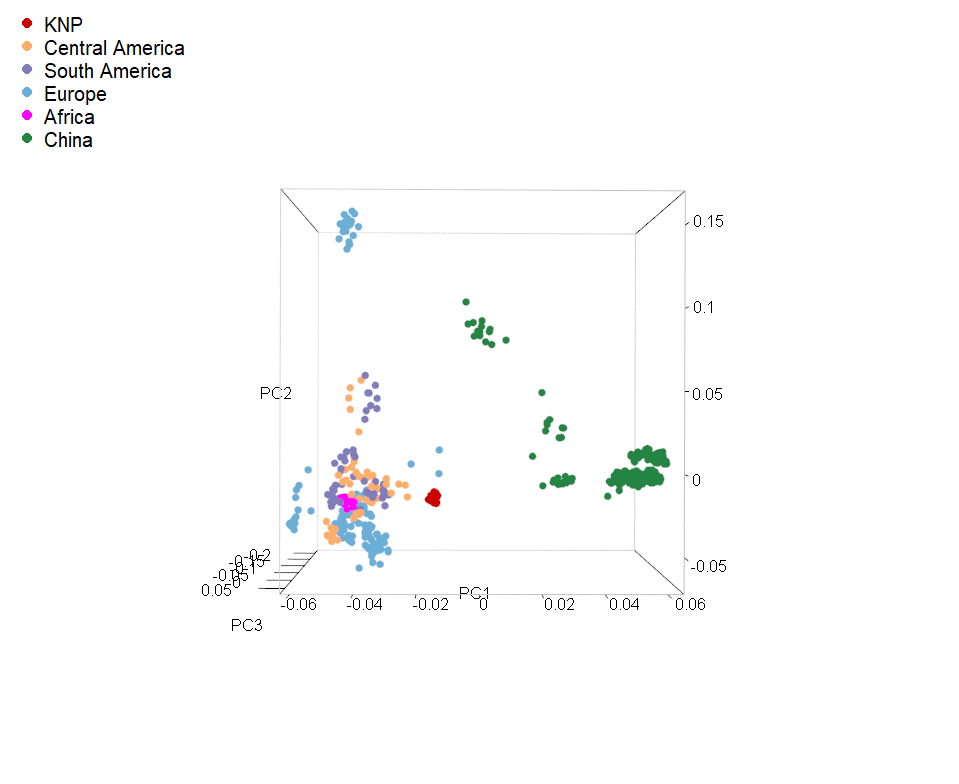

Supplement: Supplementary file 1 [file animals-10-00760-s001.zip › animals-734912-Figure S1 Visual.gif]
